# A Transitional Gundi (Rodentia: Ctenodactylidae) from the Miocene of Israel

**DOI:** 10.1371/journal.pone.0151804

**Published:** 2016-04-06

**Authors:** Raquel López-Antoñanzas, Vitaly Gutkin, Rivka Rabinovich, Ran Calvo, Aryeh Grossman

**Affiliations:** 1 School of Earth Sciences, University of Bristol, Bristol, United Kingdom; 2 Departamento de Paleobiología, Museo Nacional de Ciencias Naturales-CSIC, Madrid, Spain; 3 The Harvey M. Krueger Family Center for Nanoscience and Nanotechnology, The Hebrew University of Jerusalem, Jerusalem, Israel; 4 National Natural History Collections, Institute of Earth Sciences and Institute of Archaeology, The Hebrew University of Jerusalem, Jerusalem, Israel; 5 Geological Survey of Israel, Jerusalem, Israel; 6 Arizona College of Osteopathic Medicine, Midwestern University, Glendale, AZ, United States of America; 7 School of Human Evolution and Social Change, Arizona State University, Tempe, AZ, United States of America; Team 'Evolution of Vertebrate Dentition', FRANCE

## Abstract

We describe a new species of gundi (Rodentia: Ctenodactylidae: Ctenodactylinae), *Sayimys negevensis*, on the basis of cheek teeth from the Early Miocene of the Rotem Basin, southern Israel. The Rotem ctenodactylid differs from all known ctenodactylid species, including *Sayimys intermedius*, which was first described from the Middle Miocene of Saudi Arabia. Instead, it most resembles *Sayimys baskini* from the Early Miocene of Pakistan in characters of the m1-2 (e.g., the mesoflexid shorter than the metaflexid, the obliquely orientated hypolophid, and the presence of a strong posterolabial ledge) and the upper molars (e.g., the paraflexus that is longer than the metaflexus). However, morphological (e.g., presence of a well-developed paraflexus on unworn upper molars) and dimensional (regarding, in particular, the DP4 and M1 or M2) differences between the Rotem gundi and *Sayimys baskini* distinguish them and testify to the novelty and endemicity of the former. In its dental morphology, *Sayimys negevensis* sp. nov. shows a combination of both the ultimate apparition of key-characters and incipient features that would be maintained and strengthened in latter ctenodactylines. Thus, it is a pivotal species that bridges the gap between an array of primitive ctenodactylines and the most derived, Early Miocene and later, gundis.

## Introduction

**Fig 1:**
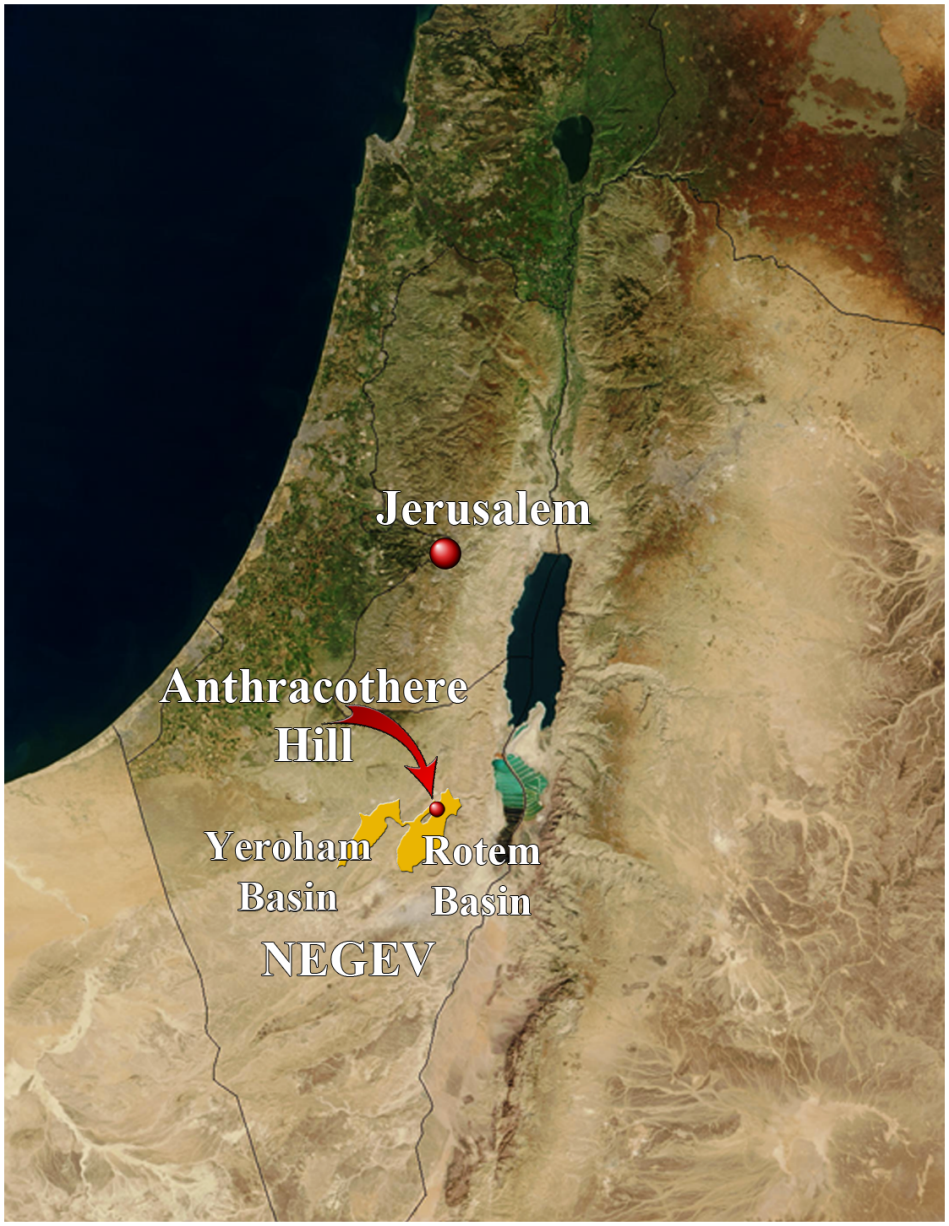
Location of Anthracothere Hill in the Rotem Basin (Israel). Moderate Resolution Imaging Spectroradiometer (MODIS) image from the Terra satellite by NASA (http://visibleearth.nasa.gov/).

**Fig 2:**
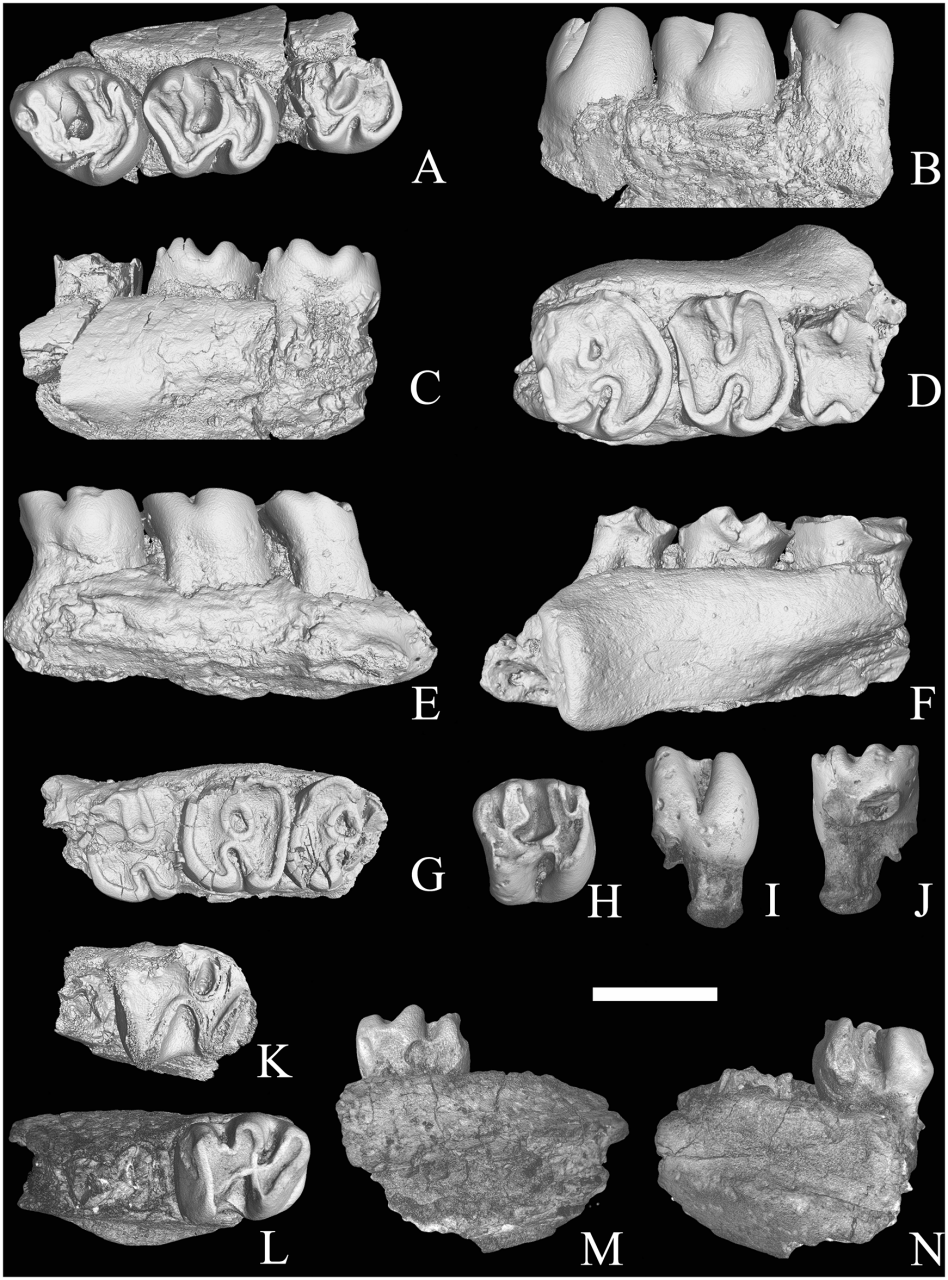
*Sayimys negevensis* sp. nov. (A-C) AH2051 (holotype), right maxilla with M1-3; (D-F) AH2143, right maxillary fragment with DP4-M2; (G) AH1792, left maxillary fragment with M1-M3; (H-J) AH1874, right M1 or M2; (K) AH2211, left mandible fragment with m1 and p4 alveolus; (L-N) AH1938, left mandible fragment with m1. (B, E, I, M) lingual views; (C, F, J, N) labial views; (A, D, G, H, K, L) occlusal views; 3D rendering from X-ray microtomography (μCT scan). Scale bar = 2 cm.

Neev [1] indicated the discovery of large mammals in the Neogene of the Rotem Basin (Israel) based on unpublished data of other geologists. Savage and Tchernov [2] mentioned again the discovery of macromammals (mostly proboscideans) in the Gidron member of the Hatzeva Formation of the Rotem and Yeroham basins and provided provisional identifications. They suggested that these fossils could be Early Burdigalian in age (MN3 equivalent 18Ma) or even older. Goldsmith et al. [3] first signaled the presence of rodent remains (such as incisors) in the Rotem Basin (see Fig 1), which they identified as: *Megapedetes* sp., *Metasayimys* sp., and *Cricetodon* sp. They also mentioned the presence of a variety of other mammals as well including birds, reptiles, and fishes ([3]: fig. 2). A few of these taxa, but no rodents, were also said to be present in the Yeroham Basin ([3]: fig. 1). The fossils were found in a site called Anthracothere Hill, which is situated on an East-facing escarpment of a North-South oriented ridge, about 100 m above the wadi in the north Rotem Basin. The fossil bearing area is situated about 60 m above the ridge base. Rodents were recovered at the lowest levels (Goldsmith et al., unpublished data). Goldsmith et al. [3] estimated the age of these fossils at 16–17 Ma (MN4 equivalent). A more detailed report was published by Tchernov et al. [4]. These authors identified the rodents from the Rotem Basin as *Megapedetes* cf. *pentadactylus*, *Metasayimys* sp., and “a probable representative of the Bathyergoidea and (?) Cricetodontidae” ([4]: 301). They suggested that the Negev fauna correlated with lower MN3. Goldsmith et al. [5] discussed the age of the mammals from the Hatzeva Formation. They considered the fauna a correlate of the upper MN3 and based also on radiometric dating of Rusinga [6], whose fauna is comparable, concluded that it is 17.5–17.0 Ma. Savage [7] discussed the age of the Negev fauna and found that it can hardly be differentiated from Gebel Zelten, which he correlated with uppermost MN3. Goldsmith et al. [8] radiometrically-dated oysters from a level a few meters above the vertebrates at 17.9 Ma. Wood and Goldsmith [9] re-examined the micromammal fauna and suggested the presence of a “new species of primitive pedetid, provisionally referred to *Megapedetes*”, *Metasayimys* cf. *intermedius*, (?) *Bathyergoides* sp., and an indeterminate species that was either a cricetid or a sciurid. As part of a study of the Miocene fauna in Israel, we re-examined the rodent material from the Rotem Basin housed at the National Natural History collections of the Hebrew University of Jerusalem. Preliminary results hinted at the presence of only a pedetid and a ctenodactylid in the early Miocene of the Rotem Basin [10]. The aim of the present paper is to describe in detail this ctenodactylid and analyse the significance of this novel taxon in its full extent.

## Material and Methods

The acronyms used in this study are: C. BR (Collection of Dr J. Braillon, Muséum national d’Histoire naturelle, Paris, France), C.G. (Catalogue général du Laboratoire des Mammifères et Oiseaux, MNHN), CM (Carnegie Museum of Natural History, Pittsburgh, USA), GSI (Geological Survey of India, Calcutta, India), FSO (Faculté des Sciences d’Oran, Algeria), Y-GSP (Yale-Geological Survey of Pakistan, Quetta, Pakistan), IVAU (Department of Earth Sciences, Utrecht, The Netherlands), MB (Museum für Naturkunde der Humboldt-Universität, Berlin, Germany), MGONM (Muséum de Géologie Office National des Mines, Tunis, Tunisia), MNHN (Muséum national d’Histoire naturelle, Paris, France), MTA (Mineral Resources and Exploration, General Directorate, Natural History Museum, Ankara, Turkey), PMNH (Pakistan Museum of Natural History, Islamabad, Pakistan), NHMR (National Heritage Museum, Riyad, Saudi Arabia); PMAE (Peabody Museum of Archaeology and Ethnology, Cambridge, USA), PMU (Palaeontological Museum, University of Uppsala, Uppsala, Sweden), PUA (Panjab University, Chandigarh, India), PIN (Paleontological Institute of the Russian Academy of Sciences, Moscow, Russia), SGM (Service Géologique du Maroc, Rabat, Morocco), UB (Üniversitat Bonn, Bonn, Germany), UM (Université de Montpellier, Montpellier, France), Z (Zinda Pir area, Pakistan).

The systematic study presented below involved the inspection of numerous specimens. We examined skulls of extant *Massoutiera mzabi* (Lataste, 1881 [11]), *Felovia vae* (Lataste, 1886 [12]), *Ctenodactylus gundi* (Rothman, 1776 [13]), *Ctenodactylus vali* Thomas, 1902 [14], *Pectinator spekei* Blyth, 1856 [15]; isolated teeth, maxillary fragments, and mandible fragments of the following extinct species: *Prosayimys flynni* Baskin, 1996 [16] from Pakistan, *Sayimys assarrarensis* López-Antoñanzas and Sen, 2004 [17] from Saudi Arabia, *Sayimys giganteus* López-Antoñanzas, Sen and Saraç, 2004 [18] from Turkey, *Sayimys intermedius* (Sen and Thomas, 1979 [19]) from Saudi Arabia and from Chios Island, Greece, *Sayimys chinjiensis* (= *Sayimys sivalensis* (Hinton, 1933 [20])) from Pakistan, *Metasayimys curvidens* Lavocat, 1961 [21] from Morocco, *Africanomys pulcher* Lavocat, 1961 [21] from Morocco. Specimen numbers and institutions are listed in S1 File.

The new specimens have been described and compared with all the valid species of Ctenodactylinae as recognized by López-Antoñanzas and Knoll [22] and a few further species erected subsequently [23, 24]. However, the detailed comparisons reported in full below are only those carried out with the species considered to be the closest relatives to the new Israeli taxon (*Prosayimys* spp. and "*Sayimys*" spp.).

First, second, and third lower molars are designated as m1, m2, and m3, respectively, and first, second, and third upper molars as M1, M2, and M3, respectively. Lower and upper permanent premolars are designated as p4 and P4, respectively, and lower and upper deciduous premolars as dp4 and DP4, respectively. The terminology used in the tooth descriptions follows the works of Baskin [16] and López-Antoñanzas and Knoll [22].

The occlusal measurements (greatest length and greatest width; Table 1) of the teeth of *Sayimys negevensis* sp. nov. from Israel have been obtained with a Nikon digital counter CM-6S measuring device.

**Table 1:** Occlusal measurements (mm) of the teeth of *Sayimys negevensis* sp. nov. All measurements represent greatest length and greatest width.

	Specimen	Length	Width
M1 or M2	AH1874	2.40	2.33
DP4	AH2143	1.71	2.07
M1	AH2143	2.12	2.50
M2	AH2143	2.12	2.34
M1	AH1792	1.79	2.00
M2	AH1792	1.86	1.97
M3 (broken)	AH1792	1.85	1.89
M1	AH2051	1.96	1.79
M2	AH2051	2.33	2.13
M3	AH2051	2.12	2.16
m1 or m2	AH1938	2.31	1.86
m1	AH2211	2.29	1.95

The evolutionary history of ctenodactyline rodents have been clarified recently [22, 25]. The analysis of the phylogenetic relationships of the Rotem gundi that we present in this work builds upon the character/taxon matrix from López-Antoñanzas et al. [25]. *Karakoromys* Matthew and Granger, 1923 [26] and *Tataromys* Matthew and Granger, 1923 [26], basal ctenodactylid genera [24, 27], were selected as the outgroup. The ingroup included all the valid species of Ctenodactylinae known to date except for those having over 50% of missing data [25]. The informative dental characters used in this work are listed in S2 File.

The data matrix (S3 File) was processed with TNT [28] with the "traditional search" option (using TBR).

### Nomenclatural acts

The electronic edition of this article conforms to the requirements of the amended International Code of Zoological Nomenclature, and hence the new name contained herein is available under that Code from the electronic edition of this article. This published work and the nomenclatural acts it contains have been registered in ZooBank, the online registration system for the ICZN. The ZooBank LSIDs (Life Science Identifiers) can be resolved and the associated information viewed through any standard web browser by appending the LSID to the prefix "http://zoobank.org/". The LSID for this publication is: urn:lsid:zoobank.org:pub:E4252CEA-BB80-4994-832A-97F70627EF80. The electronic edition of this work was published in a journal with an ISSN, and has been archived and is available from the following digital repositories: PubMed Central, LOCKSS.

## Systematic Paleontology

Order RODENTIA Bowdich, 1821 [29]

Family ctenodactylidae Gervais, 1853 [30]

Genus *SAYIMYS* Wood, 1937 [31]

*SAYIMYS NEGEVENSIS* sp. nov. (Fig 2) urn:lsid:zoobank.org:act:A9EDF03F-AF4B-49DB-865C-D91ED8C9FB19

Etymology: from the Negev desert.

Holotype: AH2051, right maxillary fragment with M1-M3 (Fig 2A–2C, S1 Fig).

Paratype: AH2143, right maxillary fragment with DP4-M2 (Fig 2D–2F); AH1792, left maxillary fragment with M1-M3 (Fig 2G); AH1874, right M1 or M2 (Fig 2H–2J); AH2211, left mandible fragment with m1 and p4 alveolus (Fig 2K); AH1938, left m1 or m2 (Fig 2L–2N); AH2048, AH 2105, AH1937, AH1229, AH2209, AH1155, AH1573, AH1973, AH1220, AH1875, AH1534, AH1551, AH1666, AH1605, AH1646, AH1807, upper incisors; AH1265, AH1346, AH1847, AH2107, AH1732, AH2097, AH1528, AH1923, lower incisors.

Repository institution: National Natural History Collections, The Hebrew University of Jerusalem, Jerusalem, Israel.

Type Locality: Anthracothere Hill, about 10 km east-southeast of Dimona, South District, Israel.

Type horizon and age: Rotem Member, Middle Hatzeva Formation; ~18 Ma (MN3-MN4 transition equivalent), Early Miocene.

Diagnosis: Ctenodactylinae with unilateral hypsodonty. Lower molars having the metalophulid II combine with the metalophulid I in an early stage of wear, a wide, open V-shaped mesoflexid shorter but deeper than the metaflexid, and a well-developed posterolabial ledge; upper molars with the paraflexus more affected by increasing wear and longer than the metaflexus. M3 slightly reduced posteriorly.

Differential Diagnosis: Distinct from *Prosayimys flynni* in the absence of a distinct metalophulid II separated from the metalophulid I on the lower molars, in having the mesoflexid shorter and deeper than the metaflexid and having a well-developed posterolabial cingulum on the lower molars. Differing from *Sayimys obliquidens* Bohlin, 1946 [32] in the absence of a distinct metalophulid II on the lower molars. Distinct from *Sayimys baskini* López-Antoñanzas and Sen, 2003 [33] and *Sayimys sivalensis* in having a well-developed paraflexus that is longer than the metaflexus, and from the latter also in having a metalophulid II and an open V-shaped mesoflexid shorter than the metaflexid. Differing from *Sayimys obliquidens*, *Sayimys assarrarensis*, and *Sayimys giganteus* in the wear pattern of the upper molars (the paraflexus is more affected by wear than the metaflexus) and from the latter also in having the M3 more posteriorly reduced. Different from *Sayimys intermedius* in having the mesoflexid shorter than the metaflexid and having a well-developed posterolabial edge on the lower molars. *Sayimys negevensis* sp. nov. differs from *Pireddamys* De Bruijn and Rümke, 1974 [34] and *Sardomys* De Bruijn and Rümke, 1974 [34] in the absence of a distinct independent metalophulid II on the lower molars. Additionally, it differs from *Sardomys* in lacking cement infilling the valleys on the lower molars. *Sayimys negevensis* sp. nov. greatly differs from that of *Irhoudia* Jaeger, 1971 [35] and *Pellegrinia* De Gregorio, 1887 [36] in the morphology of its cheek-teeth. For instance, it is much less hypsodont, lacks cement infilling in the valleys of the molars, and has four-lobed upper molars. *Sayimys negevensis* sp. nov. is also distinct from *Metasayimys* Lavocat, 1961 [21] in the absence of cement filling the valleys of the molars. Finally, the new Israeli species differs from all species of *Africanomys* Lavocat, 1961 [21] in having the M3 less reduced posteriorly.

## Description

### Lower incisors

The lower incisors are rounded and usually ungrooved.

### Lower molars

Unfortunately, only two lower molars have been found, a m1 and a m1 or m2 (AH2211 and AH1938, Fig 2K and 2L–2N, respectively). The outline of these teeth is subrectangular in occlusal view. The mesoflexid is wide, open V-shaped and clearly shorter and deeper than the metaflexid. These teeth show a remnant of metalophulid II, which is combined with the metalophulid I in a single loph (see discussion below). The hypolophid is roughly transverse and does not oppose exactly the hypoflexid. The protoconid is larger and extends more labially than the hypoconid. The posterolophid does not constrict before reaching the triangular wear surface of the hypoconid. These teeth show a low and well-developed cingulum on their posterolabial side.

### Upper incisors

The upper incisors are characterized by having a longitudinal groove located close to the labial side of the tooth.

### DP4

The only available DP4 is very badly worn (AH2143, Fig 2D–2F). Due to the large degree of dental wear of this specimen, the hypoflexus seems to be very short and the hypostria very shallow. This tooth has three roots.

### M1-2

The M1 is likely smaller than the M2. The occlusal outline of the M1 and the M2 is sub-quadrate. The protoloph connects to the medial or posterior margin of the protocone. The anteroloph and the protoloph and the metaloph and the posteroloph are fused in an early stage of wear. Therefore, the paraflexus and the metaflexus have disappeared in all specimens except for the less worn of them (AH2051 and AH1874, see Fig 2A–2C and 2H–2J respectively), in which they are well-developed. The upper molars of this new taxon seem to have a wear pattern in which the paraflexus is more affected by the increase in wear than the metaflexus and, therefore, it experiences a more rapid shortening than the metaflexus. In case of heavy wear, the paraflexus is obliterated, but even then the metaflexus may persist as an indentation. In the less worn specimens (AH2051, Fig 2A–2C and AH 1874, Fig 2H–2J), the mesoflexus extends across the tooth slightly beyond half of it and flexes posteriorly at its internal termination. The hypoflexus is shorter and much deeper than the mesoflexus and flexes anteriorly. These teeth have three roots (two labial and a lingual one).

### M3

The morphology of this tooth (AH2051) soundly recalls that of the M1 and M2 with its posterior side somewhat reduced and, therefore, with the hypocone smaller than the protocone (Fig 2A–2C).

## Comparisons

The new species from Israel is compared below with all valid species of *Prosayimys* and "*Sayimys*" (see discussion) known to date [22].

### Comparison with *Prosayimys flynni* Baskin, 1996 [16]

The holotype of this species (Z113/295) comes from the Chitarwata Formation near Dalana (Zinda Pir Dome, Pakistan) and might be latest Oligocene (ca. 23.5 Ma) in age [37]. *Sayimys negevensis* sp. nov. is more hypsodont than *Prosayimys flynni*.

**Fig 3:**
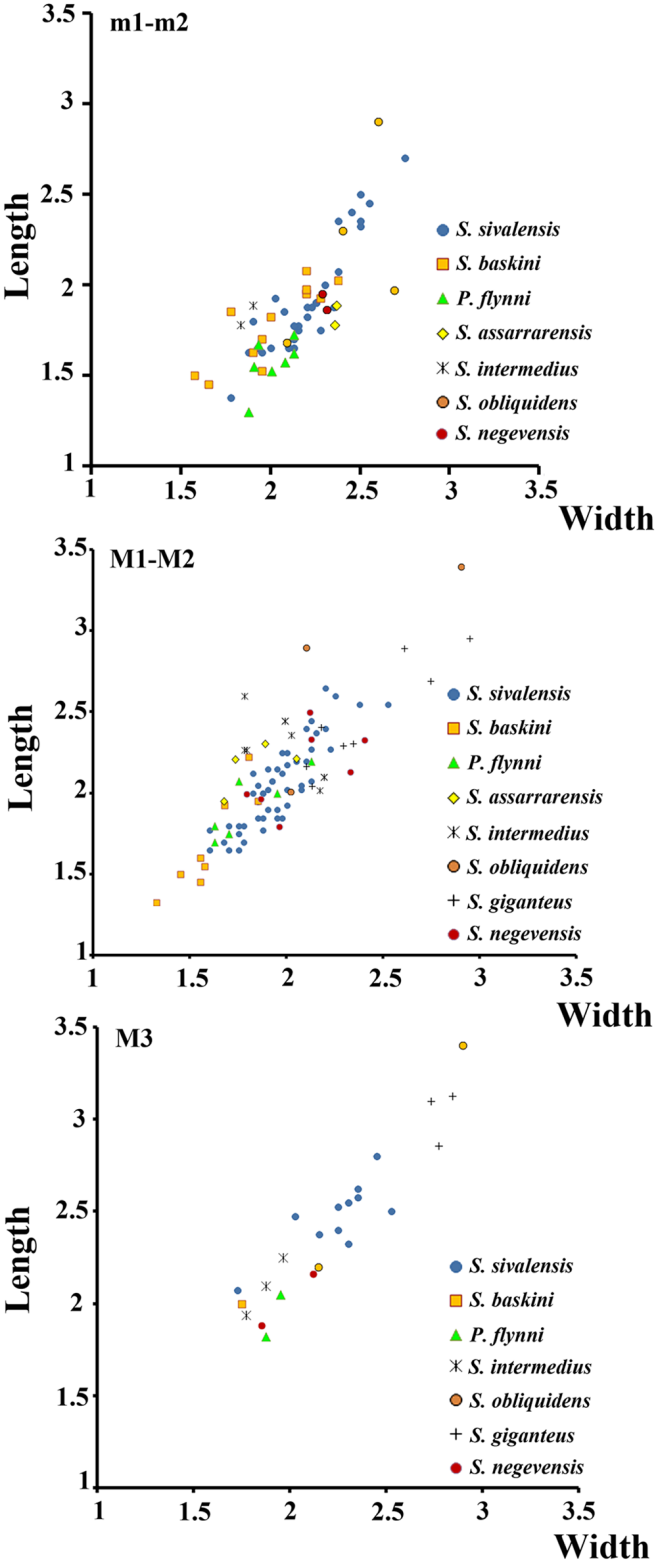
Length/width scatter diagrams of the upper molars and first and second lower molars of the species included into *Sayimys*. Red circles indicates the size of *Sayimys negevensis* sp. nov.

The teeth of *Sayimys negevensis* sp. nov. are larger than the largest equivalent teeth of *Prosayimys flynni* (Fig 3; Baskin [16, 33]).

The lower molars of *Prosayimys flynni* are easily distinguished from those of *Sayimys negevensis* sp. nov. by the presence of a distinct metalophulid II that is not fused with the metalophulid I. Furthermore, the lower molars of *Sayimys* sp. nov. have the mesoflexid shorter and deeper than the metaflexid. In contrast, those of *Prosayimys flynni* show a mesoflexid and a metaflexid nearly equal in length as well as in depth [16]. The lower molars of *Prosayimys flynni* have a poorly developed posterolabial cingulum, whereas it is well-developed in those of *Sayimys negevensis* sp. nov.

The M1-3 of *Prosayimys flynni* and *Sayimys negevensis* sp. nov show also some differences. In the upper molars of *Sayimys* sp. nov from Israel, the paraflexus and metaflexus almost disappear in an early stage of wear, whereas in *Prosayimys flynni* both persist until after very advanced wear. This is probably due to the weaker hypsodonty of the teeth of *Prosayimys*, which have deep labial flexus and lingual flexids in relation to the tooth crown height. In *Sayimys* sp. nov., the labial flexus and lingual flexids are not so deep with regard to the crown height and, therefore, are more prone to wear.

Finally, the posterior side of the M3 of *Sayimys negevensis* sp. nov. is somewhat reduced, whereas that of the M3 of *Prosayimys flynni* is not.

### Comparison with *Sayimys obliquidens* Bohlin, 1946 [32]

The holotype (T. b. 268 b) of this species ([32]:111, fig. 30b, 30b', 30b''), a left lower jaw with p4-m3, comes from an horizon of the Tiejianggou Formation in the Tabenbuluk region (Gansu, China) that is possibly Middle Miocene in age [38]. The m1-2s of *Sayimys negevensis* sp. nov. have a metalophulid II that fused with the metalophulid I early in wear, whereas those of *Sayimys obliquidens* have a distinct metalophulid II. Furthermore, the posterolophid is not constricted before reaching the triangular wear surface of the hypoconid in AH1938, whereas this constriction is observed on the m1s and m2s of *Sayimys obliquidens*. On the lower molars of *Sayimys negevensis* sp. nov., as in *Sayimys obliquidens*, the mesoflexid is much shorter than the metaflexid and the hypolophid is anteriorly directed. The comparison between the upper molars from the Rotem Basin and those belonging to *Sayimys obliquidens* are based on the single tooth row described and attributed by Bohlin ([32]: fig. 30a‴) to this species. It is necessary to note here that, as proposed implicitly by Stehlin and Schaub ([39]: fig. 182) and Schaub ([40]: fig. 212) and explicitly by Wang ([27]: 63), all of the specimens from Tabenbuluk described by Bohlin [32] possibly belong to *Sayimys obliquidens*. On the M1-2s of *Sayimys obliquidens*, the metaflexus seems to be more affected than the paraflexus by the increase in wear. In fact, in the M1 of specimen 279a ([32]: fig. 30a‴), the metaflexus is nearly obliterated, whereas the paraflexus is still preserved. In contrast, as seen above, the paraflexus on the upper molars of *Sayimys negevensis* sp. nov. is more affected by wear than the metaflexus. The upper molars described by Bohlin [32] have a mesoflexus that is much narrower than in *Sayimys negevensis* sp. nov. Both *Sayimys negevensis* sp. nov. and *Sayimys obliquidens* have the M2 larger than the M1, but all the teeth of *Sayimys obliquidens* are well over the size range of those belonging to *Sayimys negevensis* sp. nov. (Fig 3).

### Comparison with *Sayimys baskini* López-Antoñanzas and Sen, 2003 [33]

The holotype of this species, a right P4 (GSP Y747/48125), has been recorded from the Early Miocene (MN4) locality Y747 of the Kamlial Formation (Potwar Plateau, Pakistan) [33]. The morphology of the m1-2 of this taxon soundly recalls *Sayimys negevensis* sp. nov. However, the lower molars of this taxon are larger than those belonging to *Sayimys baskini* (Fig 3) and show a strong posterolabial ledge that is lacking in the latter species. The lower molars of the Rotem gundi and those of *Sayimys baskini* (Y747/48136) have a wide, open V-shaped mesoflexid shorter than the metaflexid that allows inferring the former presence of a metalophulid II (see discussion). The hypolophid of AH1938 is slightly oblique, does not oppose exactly the hypoflexid, and is partially lined up with the posterior arm of the protoconid. According to Baskin [16], the hypolophid of the m1 of GSP 48136 is more or less transverse, opposite the hypoflexid, and not aligned with the posterior arm of the protoconid. In the m2 of the same specimen, the hypolophid is more oblique and partially aligned with the posterior arm of the protoconid. As in Y747/48136, the hypoflexid is much larger and deeper than the mesoflexid in AH1938. The only DP4 of *Sayimys negevensis* sp. nov. available is badly worn. However, it is over the size range given by Baskin [16] for the DP4 of *Sayimys baskini*. The upper molars of *Sayimys negevensis* sp. nov. differ from those of *Sayimys baskini* in having a well-developed paraflexus that is longer than the metaflexus in the less worn specimens. Of the 19 M1 or M2s from the Kamlial Formation (localities Y721 and 747) described by Baskin [16], only one (GSP 36353) has a paraflexus that is longer and deeper than the metaflexus. The paraflexus is absent in the remaining teeth. Baskin [16] argued that the absence of a paraflexus is, at least in some specimens, real and not the result of wear because there are little worn specimens in which the anteroloph and/or protoloph taper(s) as it/they extend(s) labially. Furthermore, all the upper molars of the new taxon from Israel are well over the size range provided by Baskin [16, 33] for *Sayimys baskini* (Fig 3).

### Comparison with *Sayimys giganteus* López-Antoñanzas, Sen and Saraç, 2004 [18]

The holotype of this species, a fragmentary left maxilla with P4-M1, comes from the Lower Miocene (MN3-MN4) Turkish localities of Keseköy [18].

The cheek teeth of *Sayimys negevensis* sp. nov. are smaller than those of *Sayimys giganteus* (Fig 3). Moderately worn teeth of the former taxon show a pattern of three lophs due to the fusion of the anteroloph and the protoloph at an early stage of wear. Even after moderate wear, the upper molars of *Sayimys giganteus* have four very distinct lophs. In addition, the M3 of *Sayimys negevensis* sp. nov. is more reduced posteriorly than those of *Sayimys giganteus*.

### Comparison with *Sayimys assarrarensis* López-Antoñanzas and Sen, 2004 [17]

The holotype of this species (AS21–1023), a fragmentary left maxilla with P4-M2, comes from the Lower Miocene locality of As-Sarrar, Saudi Arabia [17]. In *Sayimys assarrarensis*, the paraflexus is present whatever the degree of wear of its upper molars (even when the metaflexus becomes obliterated). In contrast, the upper molars of *Sayimys negevensis* sp. nov. has the paraflexus more affected by wear than the metaflexus. In addition, the protocone and hypocone are connected by a straight endoloph, whereas it is oblique in *Sayimys negevensis* sp. nov.

### Comparison with *Sayimys intermedius* (Sen and Thomas, 1979) [19]

The holotype of this species (AJ 545) is a fragmentary left mandible with dp4-m2 from the Middle Miocene Hofuf Formation, Al Jadidah, Saudi Arabia [19].

The lower molars of *Sayimys negevensis* sp. nov. have a wide, open V-shaped mesoflexid that is shorter than the metaflexid and a well-developed posterolabial edge. In *Sayimys intermedius*, the mesoflexid and the metaflexid are equal in length and there is a weak or absent posterolabial ledge. With respect to the upper molars, those of *Sayimys negevensis* sp. nov. have the anteroloph and the protoloph fusing after moderate wear. In contrast, the upper molars of *Sayimys intermedius* show four distinct lophs even at a more advanced wear stage. In addition, the M3 of *Sayimys negevensis* sp. nov. are more reduced posteriorly than those belonging to *Sayimys intermedius*.

### Comparison with *Sayimys sivalensis* (Hinton, 1933) [20]

The holotype of *Sayimys sivalensis* (GSI D284) is a left dentary fragment with m2 and m3 from the Middle Miocene Chinji Formation, Pakistan [20]. On the m1-2s from the Israeli species, the mesoflexid is much shorter than the metaflexid and does not reach the longitudinal axis. This condition can also be observed in GSI D284, as figured by Black [41]. The specimens described as *Sayimys sivalensis* by De Bruijn et al. [42] and Baskin [16] also have a mesoflexid that is shorter than the metaflexid, but only slightly so and, in contrast with the condition in the Israeli m1-2s, it extends up to the longitudinal axis. According to Wessels et al. [43], the mesoflexid and the metaflexid of *Sayimys sivalensis* extend equally far labially in the m1, whereas the mesoflexid extends farther labially in the m2. In the specimens figured by Munthe ([44]: fig. 8C–D), the mesoflexid and metaflexid are approximately equal in length. In the lower molars of *Sayimys negevensis* sp. nov., the hypolophid is less oblique than usually observed in *Sayimys sivalensis*. In AH2299 and AH1938, the anterior arm of the hypoconid is constricted at its connection with the posterior arm of the protoconid as in the specimens of *Sayimys sivalensis*. The m1-m2 of *Sayimys negevensis* sp. nov. have a well-developed posterolabial ledge. In *Sayimys sivalensis*, this ledge is usually distinct, but it can be weak or even missing. The upper molars of *Sayimys sivalensis* have a very short paraflexus. In contrast, the M1-M3 of the less worn specimen from Israel (AH2051) have a well-developed paraflexus. The dental wear pattern of both taxa is characterized by having the paraflexus more prone to obliteration with wear than the metaflexus.

## Phylogenetic Analysis

A few attempts to decipher phylogenetic relationships in extant [45, 46] and extinct [17, 18] ctenodactylines had been carried out before López-Antoñanzas & Knoll [22] provided a comprehensive cladistics analysis of the subfamily. The subsequent discovery of a new genus of ctenodactyline from the Late Miocene of Lebanon [25] also contributed to improve our understanding of the evolutionary history of Ctenodactylinae.

The cladistic analysis including all valid species of Ctenodactylinae with less than 50% of missing data [25] has produced a single most parsimonious tree that confirms that the genus *Sayimys* is not monophyletic, as suggested by López-Antoñanzas and Knoll [22]. Vianey-Liaud et al. ([24]: fig. 15) in their interesting study of a new ctenodactylid from the Oligocene of China, *Helanshania deserta*, recovered a monophyletic Sayimyini. However, as their aim was not to resolve the relationships within the genus *Sayimys* but rather to determine the phylogenetic position of *Helanshania*, they did not include in their analysis all the species of *Sayimys* (e.g., *Sayimys obliquidens* appears in the matrix but not in the cladogram, *Sayimys assarrarensis* and *Sayimys giganteus* are lacking altogether).

**Fig 4:**
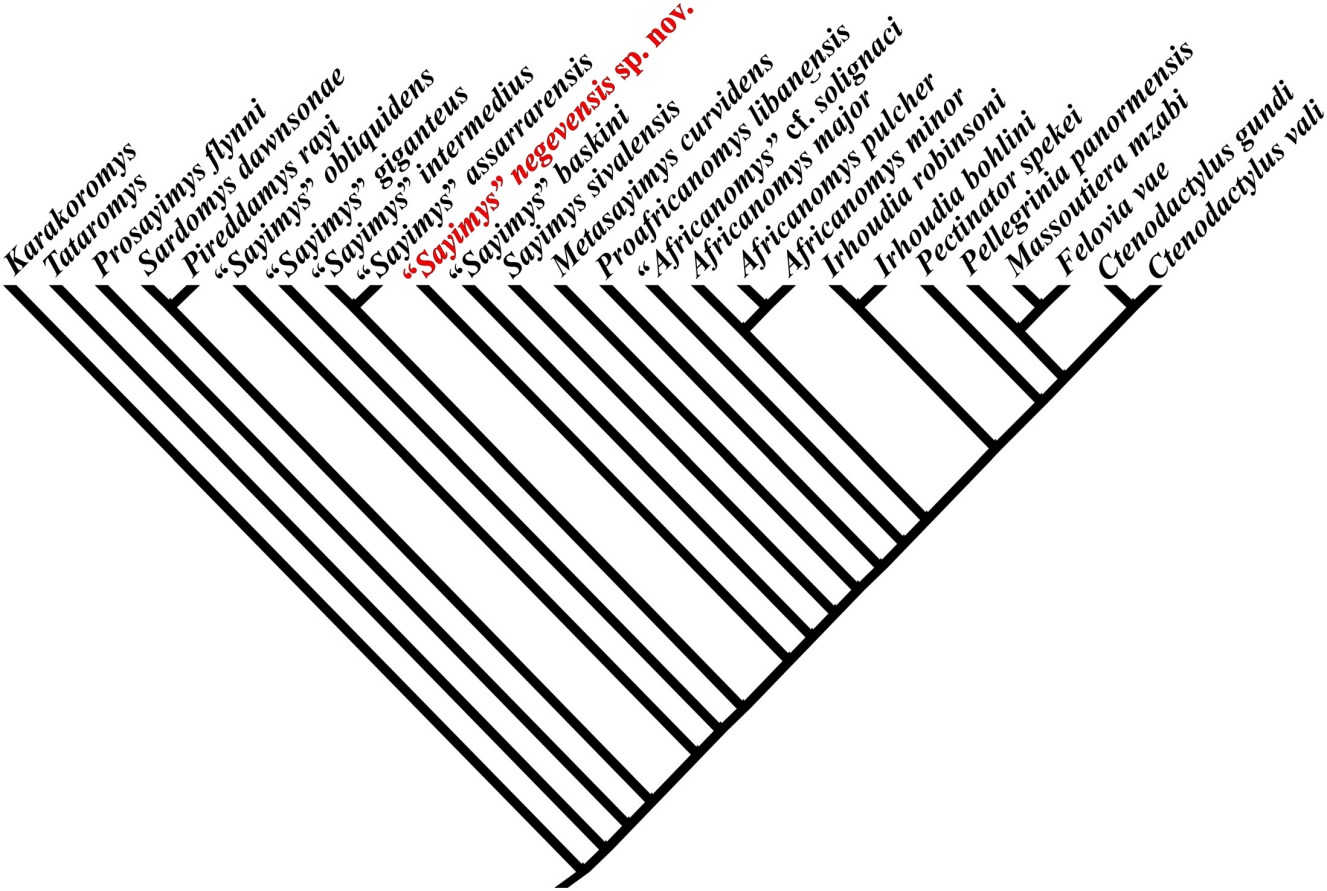
Single most parsimonious tree generated by the cladistic analysis of all valid species of the Ctenodactylinae with less than 50% of missing data [25] (matrix in S3 File). The phylogenetic position of *Sayimys negevensis* sp. nov. is highlighted in red.

The cladistic analysis including all valid species of Ctenodactylinae [25] resulted in a single most parsimonious tree (Fig 4). It shows that the Rotem gundi is more derived than the clade (*Sayimys assarrarensis* + *Sayimys intermedius*), but more primitive than *Sayimys baskini*. *Sayimys negevensis* sp. nov. shares some important synapomorphies (characters 31:1, 34:1) with the most derived taxa within Ctenodactylinae, which evidence some clear evolutionary trends inside this subfamily.

Primitive ctenodactylinae are characterized by having the paraflexus and the metaflexus on the upper molars well developed (29:0). The paraflexus is long in the most primitive ctenodactylines (*Prosayimys flynni*, *Sardomys dawsonae*, *Pireddamys rayi*, *Sayimys giganteus*, *Sayimys intermedius*, and *Sayimys assarrarensis*), whereas it obliterates very early with wear in more derived ctenodactylines (*Sayimys baskini*, *Sayimys sivalensis*, *Proafricanomys libanensis*, *Africanomys* cf. *solignaci*, *Africanomys* spp.) due to the fusion of the anteroloph and the paracone into a single loph. In fact, the most derived ctenodactylines (*Irhoudia* spp., *Pellegrinia panormensis*, and the living ctenodactylines) have completely lost it. *Sayimys negevensis* sp. nov. is the most derived ctenodactyline with a well-developed paraflexus, but this structure is lost after moderate wear. Therefore, this species can be seen as illustrating the transition within the evolution of Ctenodactylinae where the trend to lose the paraflexus is initiated.

The well-developed metaflexus (31:0) shown in primitive ctenodactylines disappears at earlier stages of wear in the course of ctenodactyline evolution until its complete disappearance in the crown group (*Pectinator spekei* + more derived ctenodactylines). *Sayimys negevensis* sp. nov. is the most plesiomorphic ctenodactyline that has clearly reduced the metaflexus on upper molars. Interestingly, this evolutionary trend is evidenced in the DP4 only higher in the tree (*Africanomys major*, *Africanomys pulcher*, *Africanomys minor* + more derived Ctenodactylinae).

The reduction of the posterior side of the M3 (34:1) characterizes all Ctenodactylinae more derived than the clade (*Sayimys assarrarensis* + *Sayimys intermedius*) and less derived than (*Irhoudia* spp. + more derived ctenodactylines) except for *Metasayimys curvidens*. Once again, *Sayimys negevensis* sp. nov. is the most primitive species within ctenodactylines to show this reduction.

On a side note, as mentioned above, the tree confirms that the genus *Sayimys* is not monophyletic. The taxa "*Sayimys*" *obliquidens*, "*Sayimys*" *giganteus*, "*Sayimys*" *assarrarensis*, and "*Sayimys*" *intermedius* are not closely related to *Sayimys sivalensis*, the senior synonym of the type species, *Sayimys perplexus* [22]. In addition, *Sayimys sivalensis* has numerous apomorphies that are not shared by any of the above mentioned species (characters: 27:2, 28:2, 29:1, 31:1, and 34:1). Thus, "*Sayimys*" *obliquidens*, "*Sayimys*" *giganteus*, "*Sayimys*" *assarrarensis*, and "*Sayimys*" *intermedius* cannot be considered any longer as belonging to the genus *Sayimys*. *Sayimys negevensis* sp. nov. records the appearance, at least in inchoate form, of the synapomorphies that characterize the more derived ctenodactylines. *Sayimys negevensis* sp. nov. is very close to *Sayimys baskini*, which is, in turn, very close to *Sayimys sivalensis*. The unique combination of plesiomorphic and apomorphic characters of this taxon suggests that the erection of a new genus would be warranted. However, we refrain from doing so in view of the missing data due to the current lack of premolars. The same holds true for *Sayimys baskini*, which is located phylogenetically between *Sayimys negevensis* sp. nov. and *Sayimys sivalensis*. The acquisition of new data about these species of gundis may result in a shift of their phylogenetic position.

## Conclusion

The Rotem ctenodactyline, which was assigned originally to "*Metasayimys*" cf. *intermedius* [9], can be distinguished from *Sayimys intermedius* and assigned to a new species, *Sayimys negevensis*. Compared to *Sayimys intermedius*, the lower molars of *Sayimys negevensis* have the mesoflexid shorter than the metaflexid and have a well-developed posterolabial edge. Furthermore, the anteroloph and the protoloph on the upper molars of the new taxon fuse at a moderate stage of wear and the M3 is reduced posteriorly, whereas in *Sayimys intermedius* the upper molars show four distinct lophs and the M3 is not reduced posteriorly. The taxon from Israel resembles *Sayimys baskini* from the Early Miocene of Pakistan. However, its less worn upper molars (Fig 2A and 2B) have a well-developed paraflexus, which is absent in *Sayimys baskini*. Thus, the morphological and dimensional features of the Rotem gundi show that it represent a new, endemic species. *Sayimys negevensis* nov. sp. places phylogenetically between (*Sayimys intermedius*, *Sayimys assarrarensis*) and *Sayimys baskini*. *Sayimys negevensis* sp. nov. adds a new facet to our currently poor knowledge of Middle Eastern Miocene rodents. This holds especially true for the Levant, which is an area of particular paleogeographical significance that, since Early Miocene time, has acted as a corridor of dispersal between Eurasia and Africa and where no other site of this age yielding rodents has so far been discovered.

## Supporting Information

S1 Fig Surface rendering of the holotype (AH2051) of *Sayimys negevensis* sp. nov. (PDF)

S1 File Specimen numbers, origins and housing institutions of the extinct and extant ctenodactylines examined in this work. (DOCX)

S2 File Characters used in the phylogenetic analysis. Seventeen characters are binary and seventeen are multistate. The polarity of characters was determined by outgroup comparison.
(DOCX)

S3 File Character/taxon matrix used in this work [25]. Character scoring: 0, 1, and 2, conditions of character;?, character state uncertain.
(DOC)

## References

[1] Neev D (1960) A pre-Neogene erosion channel in the southern coastal region of Israel. Bull. Geol Surv Israel 25:1–21.

[2] Savage RJG, Tchernov E (1968) Miocene mammals of Israel. Proc Geol Soc London 1648: 98–101.

[3] Goldsmith NF, Tchernov E, Ginsburg L, Tassy P, van Couvering JA (1982) Ctenodactylid rodents in the Miocene Negev fauna of Israel. Nature 296: 645–647.

[4] Tchernov E, Ginsburg L, Tassy P, Goldsmith NF (1987) Miocene mammals of the Negev (Israel). J Vertebr Paleontol 7: 284–310.

[5] Goldsmith NF, Hirsch F, Friedman GM, Tchernov E, Derin B, Gerry E, Horowitz A, Weinberger G (1988) Rotem mammals and Yeroham crassostreids: stratigraphy of the Hazeva Formation (Israel) and the paleogeography of Miocene Africa. Newsl Stratigr 20:73–90.

[6] Van Couvering JA, Miller JA (1969) Miocene stratigraphy and age determinations, Rusinga Island, Kenya. Nature 221:628–632.

[7] Savage RJG (1990) The African dimension in European Early Miocene mammal faunas In: Lindsay EH, Falhbusch V, Mein P, eds. European Neogene Mammal Chronology. New York: Plenum Press 587–599.

[8] Goldsmith NF, Martinell J, Demarco G, Bohn-Havas M, Dockery DT (1994) Sr-isotopic calibration of Cenozoic bivalvia and Early Miocene migrations: Eurasian carnivores to Africa (the Hazeva Formation, Israel) and African gazelles and proboscidia to Ipolytarrnoc, Hungary. Newsl Stratigr 31: 167–183.

[9] Wood AE, Goldsmith NF (1998) Early Miocene rodents and lagomorphs from Israel. J Vertebr Paleontol 18 (Suppl): 87A–88A.

[10] López-Antoñanzas R, Gutkin V, Rabinovich R, Calvo R, Grossman A (2014) The rodent fauna from the Early Miocene of the Rotem Basin (Israel): African, Asian, both or neither? J Vertebr Paleontol 34 (Suppl): 170.

[11] Lataste F (1881) Sur un rongeur nouveau du Sahara algérien (Ctenodactylus mzabi n. sp.). Bull Soc zool Fr 6: 214–225.

[12] Lataste F (1886) Novi subgeneris et novæ speciei rodentium, e genere *Massoutiera* diagnoses. Naturaliste 36: 287.

[13] Rothman G (1776) Reise nach Garean, im Gebiete von Tripoli, im Novemb. und Decemb. 1774: Ein Schreiben an den Ritter Wargentin in Stockholm In: Schlözer AL, ed. Neuer Briefwechsel historischen und politischen Inhalts. Heft 5. Göttingen: Vandenhoek 326–342.

[14] Thomas O (1902) On the mammals collected during the Whitaker Expedition to Tripoli. Proc Zool Soc London 2: 2–13.

[15] Blyth E (1856) Report on a zoological collection from the Somali country. J Asiat Soc Bengal 24: 291–306.

[16] Baskin JA (1996) Systematic revision of Ctenodactylidae (Mammalia, Rodentia) from the Miocene of Pakistan. Palaeovertebrata 25: 1–49.

[17] López-Antoñanzas R, Sen S (2004) Ctenodactylids from the Lower and Middle Miocene of Saudi Arabia. Palaeontology 47: 1477–1494.

[18] López-Antoñanzas R, Sen S, Saraç G (2004) A new large ctenodactylid species from the Lower Miocene of Turkey. J Vertebr Paleontol 24: 676–688.

[19] Sen S, Thomas H (1979) Découverte de rongeurs dans le Miocène moyen de la Formation Hofuf (Province du Hasa, Arabie Saoudite). C R Somm Soc géol Fr 21: 34–37.

[20] Hinton MAC (1933) Diagnoses of new genera and species of rodents from Indian Tertiary deposits. Ann Mag Nat Hist 12: 620–622.

[21] Lavocat R (1961) Le gisement de vertébrés miocènes de Beni Mellal (Maroc): étude systématique de la faune de mammifères. Notes Mém Serv géol Maroc 155: 29–77.

[22] López-Antoñanzas R, Knoll F (2011) A comprehensive phylogeny of the gundis (Ctenodactylinae, Ctenodactylidae). J Syst Paleontol 9: 379–398.

[23] Mein P, Pickford M (2010) Vallesian rodents from Sheikh Abdallah, Western Desert, Egypt. Hist Biol 22: 224–259.

[24] Vianey-Liaud M, Gomes-Rodrigues H, Marivaux L (2010) A new Oligocene Ctenodactylinae (Rodentia: Mammalia) from Ulantatal (nei Mongol): new insight on the phylogenetic origins of the modern Ctenodactylidae. Zool J Linn Soc 160: 531–550.

[25] López-Antoñanzas R, Knoll F, Maksoud S, Azar D (2015) First Miocene rodent from Lebanon provides the missing link between Asian and African gundis (Rodentia: Ctenodactylidae). Sci Rep. 5: 12871; doi: 10.1038/srep12871 26250050 10.1038/srep12871 PMC4528195

[26] Matthew WD, Granger W (1923) Nine new rodents from the Oligocene of Mongolia. Am Mus Novit 102: 1–10.

[27] Wang BY (1997) The mid-Tertiary Ctenodactylidae (Rodentia, Mammalia) of Eastern and Central Asia. Bull Am Mus Nat Hist 234: 1–88.

[28] Goloboff P, Farris J, Nixon KC (2008) TNT, a free program for phylogenetic analysis. Cladistics 24: 774–786.

[29] Bowditch TE (1821) An analysis of the natural classifications of Mammalia for the use of students and travellers. Paris: J Smith 115 p.

[30] Gervais MP (1853) Description ostéologique de l’*Anomalurus* et remarques sur la classification naturelle des rongeurs. Ann Sci nat Zool 20: 238–246.

[31] Wood AE (1937) Fossil rodents from the Siwalik beds of India. Am J Sci 34: 64–76.

[32] Bohlin B (1946) The fossil mammals from the Tertiary deposits of Taben-Baluk, Western Kansu, Part II: Simplicidentata, Carnivora, Artiodactyla, Perissodactyla, and Primates. Palaeont Sinica C 8b: 1–259.

[33] López-Antoñanzas R, Sen S (2003) Systematic revision of Mio-Pliocene Ctenodactylidae (Mammalia, Rodentia) from the Indian subcontinent. Eclogae Geol Helv 96: 521–529.

[34] De Bruijn H, Rümke CG (1974) On a peculiar association from the Miocene of Oschiri (Sardinia). Proc Kon Nederl Akad Wetensch B 77: 45–79.

[35] Jaeger JJ (1971) Un cténodactylidé (Mammalia, Rodentia) nouveau, Irhoudia bohlini n.g. n. sp. du Pléistocène inférieur du Maroc, rapports avec les formes actuelles et fossiles. Notes Mém Serv géol Maroc 31: 113–140.

[36] De Gregorio A (1887) Intorno a un deposito di roditori e di carnivori sulla vetta di Monte Pellegrino. Atti Soc Tosc Sci Nat Pisa 8: 3–39.

[37] Flynn LJ, Lindsay EH, Pilbeam D, Raza SM, Morgan ME, Barry JC, Badgley CE, Behrensmeyer AK, Cheema IU, Rajpar AR, Opdyke ND (2013) The Siwaliks and Neogene evolutionary biology in South Asia In: Wang X, Flynn LJ, Fortelius M, eds. Fossil Mammals of Asia: Neogene biostratigraphy and chronology. New York: Columbia University Press 353–372.

[38] Wang XM, Li Q, Qiu ZD, Xie GP, Wang BY, Qiu ZX, Tseng ZJ, Takeuchi GT, Deng T (2013) Neogene mammalian biostratigraphy and geochronology of the Tibetan Plateau In: Wang X, Flynn LJ, Fortelius M, eds. Fossil Mammals of Asia: Neogene biostratigraphy and chronology. New York: Columbia University Press 274–292.

[39] Stehlin HG, Schaub S (1951) Die Trigonodontie der simplicidentaten Nager. Schweiz Palaeontol Abh 67: 1–385.

[40] Schaub S (1958) Simplicidentata (= Rodentia) In: Piveteau J, ed. Traité de Paléontologie. Vol. 6 (2). Paris: Masson et Cie 659–818.

[41] Black CC (1972) Review of fossil rodents from the Neogene Siwalik Beds of India and Pakistan. Palaeontology 15: 238–266.

[42] De Bruijn H, Boon E, Hussain ST (1989) Evolutionary trends in Sayimys (Ctenodactylidae, Rodentia) from the Lower Manchar Formation (Sind, Pakistan). Proc Kon Nederl Akad Wetensch B 92: 191–214.

[43] Wessels W, de Bruijn H, Hussain ST, Leinders J (1982) Fossil rodents from the Chinji Formation, Banda Daud Shah, Kohat, Pakistan. Proc Kon Nederl Akad Wetensch B 85:337–364.

[44] Munthe J (1980) Rodents of the Miocene Daud Khel Local Fauna, Mianwali District, Pakistan. Part 1. Sciuridae, Gliridae, Ctenodactylidae, and Rhizomyidae. Milwaukee Pub Mus Contr Biol Geol 34: 1–36.

[45] George W. (1979). The chromosomes of the hystricomorphous family Ctenodactylidae (Rodentia:? Sciuromorpha) and their bearing on the relationships of the four living genera. Zool J Linn Soc 65: 261–280.

[46] George W. (1985). Cluster analysis and phylogenetics of five species of Ctenodactylidae (Rodentia). Mammalia 49: 53–63.

