# Supplementary figures and images for: A Transitional Gundi (Rodentia: Ctenodactylidae) from the Miocene of Israel
Source: PLoS One. 2016 Apr 6;11(4):e0151804. doi: 10.1371/journal.pone.0151804 (PMC4822958; doi:10.1371/journal.pone.0151804)

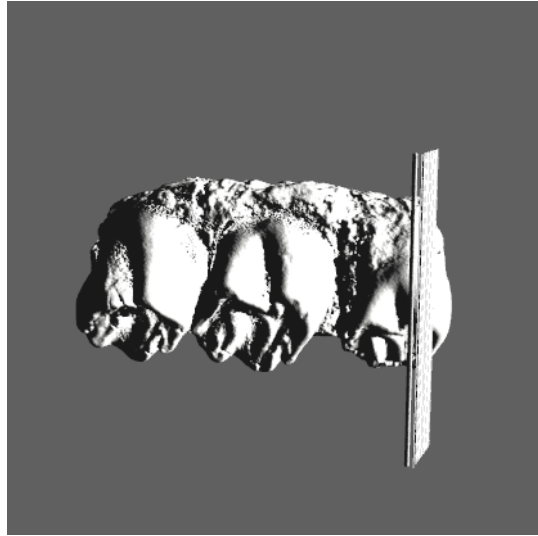

Supplement: S1 Fig — (PDF) [file pone.0151804.s001.pdf]
