# Supplementary material for: A Transitional Gundi (Rodentia: Ctenodactylidae) from the Miocene of Israel
Source: PLoS One. 2016 Apr 6;11(4):e0151804. doi: 10.1371/journal.pone.0151804 (PMC4822958; doi:10.1371/journal.pone.0151804)
Supplement: S1 File — (DOCX) [file pone.0151804.s002.docx]

**SUPPORTING INFORMATION**

**A transitional gundi (Rodentia: Ctenodactylidae) from the Miocene of Israel**

**Raquel López-Antoñanzas^1,2^*, Vitaly Gutkin^3^, Rivka Rabinovich^4^, Ran Calvo^5^, Aryeh Grossman^6,7^**

**File S1** The systematic study presented in this work involved the inspection of numerous specimens. We examined the following: skulls of extant *Massoutiera mzabi* (Lataste, 1881 [12]) (71064, 71149, 71154, 71156, 71157, 71152, 71155, 71150,71158, 71161, 71163, 71164, 71151, 71162, 71159, 37735, 37736, 37737, 37738, 37739 in the MB and C.G.1960-3741, C.G.1959-93, C.G.1960-3812, C.G.1959-92, C.G.1989-29, C.G.1912-322, C.G.1953-381, C.G.1955-3, C.G.2000-686 in the MNHN), *Felovia vae* (Lataste, 1886 [13]) (41239, 41242, 4124 in the MB and CG-1994-612, CG-1994-613, C.G.1995-3157 in the MNHN), *Ctenodactylus gundi* (Rothman, 1776 [14]) (15515, 15516, 71186, 25640, 37765, 1302, 71177, 71188, 71185, 20721, 2784, 71179, 71181, 71187, 71182 in the MB and C.G.1963-921, C.G.1975-303, C.G.1975-304, C.G.1975-305, C.G.1975-306, C.G.1975-307, C.G.1975-309, C.G.1975-308, C.G.1975-306, C.G.1975-304, C.G.1905-437, C.G.1991-1298, C.G.1991-1316, C.G.1993-1680, C.G.2007-329 in the MNHN), *Ctenodactylus vali* Thomas, 1902 [15] (C.G.1952-664, C.G.1952-666, C.G.1952-668, C.G.1951-389, C.G.1953-787 in the MNHN), *Pectinator spekei* Blyth, 1856 [16] (26563, 71171, 71169, 71153, 37977, A2636, 3935, 71165, 71166, 71167, 71175, 71170, 71172, 71174, 71173 in the MB and C.G.1895-461, C.G.1895-459, C.G.1895-460, C.G.1986-240, C.G.1978-263, C.G.1978-264, C.G.1978-265, C.G.1978-266, C.G.1978-267, C.G.1981-504, C.G.1960-3744, C.G.1960-3783 in the MNHN); isolated teeth, maxillary fragments, and mandible fragments of the following extinct species: *Prosayimys* *flynni* Baskin, 1996 [17] (casts of Z295, Z307, Z317, Z312, Z308, Z309, Z311, Z313, Z310, Z316, Z294, Z292, Z287, Z289, Z293, Z290, Z306, Z296, Z291, Z305, Z288, Z303, Z304, Z297 in RLA’s personal collection, the original material of this species is currently housed at the PMAE until it is deposited at the PMNH, *Sayimys assarrarensis* López-Antoñanzas and Sen, 2004 [18] from Saudi Arabia (AS21-1023, AS21 1001, AS21 1002, AS8 1001, AS21 1008, AS8 1000, AS21 1005, AS21 1004, AS21 1018, AS21 1017, AS8 1003, AS21 1016, AS21 1024, AS21 1026, AS21 1028, AS8 1002, AS21 1025 in the MNHN), *Sayimys* *giganteus* López-Antoñanzas, Sen and Saraç, 2004 [19] from Turkey (KSK1-100 to KSK1-102; KSK2-100 to KSK2-104; HJ-100 to HJ-108 in the MNHN), *Sayimys* *intermedius* (Sen and Thomas, 1979 [20]) from Saudi Arabia (TMA 100, TMA 101 in the MNHN) and from Chios Island, Greece (THA91-01 to THA91-03, THA91-10 to THA91-14, THA91-21 to THA91-25, THA91-28 to THA91-31; THA93-26, THA93-27, THA93-04 to THA93-09; THA93-15 to THA93-20, THA93-32 to THA93-36 in the MNHN), *Sayimys* *chinjiensis* (= *Sayimys* *sivalensis* (Hinton, 1933 [21])) from Pakistan (casts of Y-GSP 634/45186, Y-GSP 634/45183, Y-GSP 634/45187 in RLA’s personal collection), *Metasayimys curvidens* Lavocat, 1961 [22] from Morocco (Ben Mel 1357, Ben Mel 1353, Ben Mel 1354, Ben Mel 1371, CBR-188, CBR-189, CBR-191, CBR-192, CBR-194, CBR-197, CBR-198, CBR-199, CBR-205, CBR-207, CBR-208, CBR-209, CBR-210, CBR-211, CBR-212, CBR-214, CBR-215, CBR-218-CBR-222, CBR-225-CBR-230, CBR-234-CBR-236, CBR-238, CBR-248, CBR-249, CBR-251, CBR-252, CBR-257, CBR-261, CBR-268, CBR-271, CBR-274, CBR-281-CBR-283, CBR-287, CBR-289, CBR-291, CBR-294, CBR-296, CBR-300- CBR-302, CBR-304-CBR-309, CBR-311, CBR-315-CBR-328, CBR-331, CBR-335, CBR-337, CBR-338, CBR-347, CBR-350, CBR-352, CBR-356-CBR-359, CBR-360, CBR-363, CBR-370, CBR-373-CBR-384, CBR-387-CBR-389, CBR-396, CBR-398, CBR-399, CBR-460-CBR-462, CBR-465, CBR-469, CBR-471-CBR-475, CBR-477-CBR-479, CBR-481, CBR-484, CBR-487, CBR-488, CBR-531, CBR-534, CBR-535, CBR-537, CBR-551, CBR-553-CBR-556, CBR-558, CBR-559, CBR-562, CBR-565, CBR-566, CBR-568, CBR-571-CBR-574 CBR-577, CBR-578, CBR-580, CBR-696, CBR-697 in the MNHN), *Africanomys pulcher* Lavocat, 1961 [22] from Morocco (Ben Mel 1356; Ben Mel 1367, Ben Mel 1369, CBR-2-CBR-5, CBR-7-CBR-10, CBR-12, CBR-13, CBR-16, CBR-19, CBR-23, CBR-27, CBR-28, CBR-30, CBR-49-CBR-57, CBR-59, CBR-61, CBR-62-CBR-65, CBR-66, CBR-68, CBR-69-CBR-72-CBR-74, CBR-76-CBR-78, CBR-84-CBR-87, CBR-89, CBR-90, CBR-91, CBR-96- CBR-98, CBR-101, CBR-106, CBR-107, CBR-113, CBR-116, CBR-117, CBR-127-CBR-135, CBR-144, CBR-145, CBR-147-CBR-151, CBR-153-CBR-164, CBR-166, CBR-168, CBR-169, CBR-170, CBR-172, CBR-174, CBR-180, CBR-181, unnumbered CBR in the MNHN).
