# Supplementary material for: A Transitional Gundi (Rodentia: Ctenodactylidae) from the Miocene of Israel
Source: PLoS One. 2016 Apr 6;11(4):e0151804. doi: 10.1371/journal.pone.0151804 (PMC4822958; doi:10.1371/journal.pone.0151804)
Supplement: S2 File — Seventeen characters are binary and seventeen are multistate. The polarity of characters was determined by outgroup comparison. (DOCX) [file pone.0151804.s003.docx]

**SUPPORTING INFORMATION**

**A transitional gundi (Rodentia: Ctenodactylidae) from the Miocene of Israel**

**Raquel López-Antoñanzas^1,2^*, Vitaly Gutkin^3^, Rivka Rabinovich^4^, Ran Calvo^5^, Aryeh Grossman^6,7^**

**File S2**

1. LM1: (0) 1-2.5mm; (1) 2.5-3.5mm; (2) >3.5mm.
2. (0) Rooted cheek teeth; (1) ever-growing cheek teeth.
3. (0) Brachyodont; (1) mesodont; (2) hypsodont.
4. p3: (0) present; (1) absent.

dp4

1. (0) With three main lophs; (1) bilobed.
2. (0) With metalophulid II; (1) with differentiated metalophulid II and metalophulid I; (2) with combined metalophulid II and metalophulid I; (3) only with metalophulid I.
3. Anteroconid: (0) absent; (1) present.
4. Metaconid: (0) anterolingual; (1) lingual; (2) anterior.
5. Metaconid: (0) connected to the metalophulid II; (1) connected to the metalophulid I.
6. Posterolophid: (0) long; (1) shortened; (2) fused with the entoconid/hypolophid forming a single lobe.

p4

1. (0) with entoconid and hypoconid reduced to a cingulid; (1) with only a posterior cuspid (entoconid); (2) minute.
2. Shape in occlusal view (0) U-shaped pattern; (1) Y-shaped pattern.
3. Posterolabial ledge: (0) absent; (1) present.

m1-m2

1. Unworn teeth with: (0) four lobes; (1) three lobes; (2) two lobes.
2. Metalophulid II: (0) present; (1) in early wear fused with metalophulid I; (2) absent.
3. Mesoflexid (labiolingually): (0) shorter than the metaflexid; (1) equal or longer than the metaflexid.
4. Metaflexid (labiolingually): (0) long; (1) short; (2) absent.
5. Posterolabial ledge: (0) absent; (1) present.
6. Hypertrophy of the protoconid: (0) no; (1) yes.
7. Cement (on the m2-m3): (0) absent; (1) present.
8. (0) m2 longer than m3; (1) m2 equal in length with m3; (2) m2 shorter than m3.
9. Posterolophid: (0) well-developed; (1) absent.

I1

1. Groove on the upper incisor: (0) present; (1) absent.

DP4

1. Metaflexus: (0) long; (1) short; (2) absent in early wear.
2. Paraflexus: (0) long; (1) short; (2) absent in early wear.

P4

1. (0) as wide as or wider than M1; (1) reduced but with small synclines; (2) with vestigial or without synclines; (3) minute; (4) absent.
2. (0) with vestigial anteroloph, protoloph, metaloph, and posteroloph but with protoloph and metaloph still distinct; (1) anteroloph short and one connection between metacone and protocone; (2) anteroloph absent, and one connection between metacone and protocone.
3. (0) with vestigial anteroloph, protoloph, metaloph, and posteroloph but with protoloph and metaloph still distinct; (1) posteroloph short and one connection between metacone and protocone; (2) anteroloph and posteroloph absent, and one connection between metacone and protocone.

M1-M2

1. Paraflexus: (0) well developed; (1) fused in early wear; (2) absent.
2. Hypertrophy of the protocone: (0) no; (1) yes.
3. Metaflexus: (0) long; (1) short; (2) fused in early wear; (3) absent.
4. Hypoflexus: (0) deep; (1) shallow; (2) absent.
5. Cement: (0) absent; (1) present.

M3

1. labiolingual length of the posterior lobe: (0) not reduced; (1) reduced.

|  | **1** | **2** | **3** | **4** | **5** | **6** | **7** | **8** | **9** | **10** | **11** | **12** | **13** | **14** | **15** | **16** | **17** | **18** | **19** | **20** | **21** | **22** | **23** | **24** | **25** | **26** | **27** | **28** | **29** | **30** | **31** | **32** | **33** | **34** |
| --- | --- | --- | --- | --- | --- | --- | --- | --- | --- | --- | --- | --- | --- | --- | --- | --- | --- | --- | --- | --- | --- | --- | --- | --- | --- | --- | --- | --- | --- | --- | --- | --- | --- | --- |
| *Karakoromys* | 0 | 0 | 0 | 0 | 0 | 0 | 0 | 0 | 0 | 0 | 0 | 0 | 0 | 0 | 0 | 0 | 0 | 0 | 0 | 0 | 0&1&2 | 0 | 0 | 0 | 0 | 0 | 0 | 0 | 0 | 0 | 0 | 1 | 0 | 1 |
| *Tataromys* | 2 | 0 | 0 | 0 | 0 | 0 | 0 | 0 | 0 | 0 | 0 | 0 | 0 | 0 | 0 | 0 | 0 | 0 | 0 | 0 | 0 | 0 | 0 | 0 | 0 | 0 | 0 | 0 | 0 | 0 | 0 | 1 | 0 | 0 |
| *Prosayimys flynni* | 0 | 0 | 0 | ? | 0 | 1 | 1 | 1 | 1 | 0 | 1 | 0 | 0 | 0 | 0 | 0 | 0 | 1 | 0 | 0 | 1 | 0 | ? | 0 | 0 | 1 | 1 | 1 | 0 | 0 | 0 | 0 | 0 | 0 |
| *Sardomys dawsonae* | 2 | 0 | 1 | ? | 0 | 1 | 1 | 1 | 1 | 0 | 1 | 1 | 1 | 0 | 0 | 0 | 0 | 1 | 0 | 0 | 2 | 0 | 0 | ? | ? | ? | ? | ? | 0 | 0 | 0 | 0 | 0 | 0 |
| *Pireddamys rayi* | 2 | 0 | 1 | ? | ? | ? | ? | 1 | ? | ? | ? | ? | ? | 0 | 0 | 0 | 0 | 1 | 0 | 0 | 2 | 0 | 0 | ? | ? | 2 | 2 | 2 | 0 | 0 | 0 | 0 | 0 | ? |
| *Sayimys obliquidens* | 0 | 0 | 1 | ? | 0 | 1 | 1 | 1 | 1 | 0 | 1 | 1 | 1 | 0 | 0 | 0 | 0 | 1 | 0 | 0 | 1 | 0 | 0 | 0 | 0 | 1 | 1 | 1 | 0 | 0 | 0 | 0 | 0 | 0 |
| *Sayimys giganteus* | 1 | 0 | 1 | 0 | 0 | 2 | 1 | 1 | 1 | 0 | 1 | 1 | 1 | 1 | 1 | 0 | 0 | 1 | 0 | 0 | 1 | 0 | ? | 0 | 0 | 1 | 1 | 1 | 0 | 0 | 0 | 0 | 0 | 0 |
| *Sayimys baskini* | 0 | 0 | 1 | ? | 0 | 2 | 1 | 1 | 1 | 0 | 1 | 1 | 1 | 1 | 1 | 0 | 0 | 1 | 0 | 0 | 2 | 0 | ? | 0 | 0 | 1 | 2 | 1 | 1 | 0 | 1 | 0 | 0 | 1 |
| *Sayimys intermedius* | 0 | 0 | 1 | 0 | 0 | 3 | 1 | 1 | 1 | 0 | 1 | 1 | 1 | 1 | 2 | 1 | 0 | 0&1 | 0 | 0 | 1 | 0 | ? | 0 | 0 | 1 | 1 | 1 | 0 | 0 | 0 | 0 | 0 | 0 |
| *Sayimys assarrarensis* | 0 | 0 | 1 | ? | 0 | 2 | 1 | 1 | 1 | 0 | 1 | 1 | 1 | 1 | 1 | 0 | 0 | 1 | 0 | 0 | ? | 0 | ? | 0 | 0 | 1 | 1 | 1 | 0 | 0 | 0 | 0 | 0 | ? |
| *Sayimys sivalensis* | 0 | 0 | 1 | ? | 0 | 3 | 1 | 1 | 1 | 0 | 1 | 1 | 1 | 1 | 2 | 1 | 0 | 0&1 | 0 | 0 | 2 | 0 | ? | 0 | 0 | 1 | 2 | 2 | 1 | 0 | 1 | 0 | 0 | 1 |
| *Sayimys negevensis* | 0 | 0 | 1 | ? | ? | ? | ? | ? | ? | ? | ? | ? | ? | 1 | 1 | 0 | 0 | 1 | 0 | 0 | ? | 0 | 0 | ? | ? | ? | ? | ? | 0 | 0 | 1 | 0 | 0 | 1 |
| *Metasayimys curvidens* | 0 | 0 | 1 | 0 | 0 | 2&3 | 1 | 1 | 1 | 0 | 1 | 1 | 0 | 1 | 1 | 0 | 0 | 1 | 0 | 1 | 2 | 0 | 0 | 0 | 0 | 3 | 2 | 2 | 1 | 0 | 2 | 0 | 1 | 0 |
| *Africanomys pulcher* | 0 | 0 | 1 | 0 | 0 | 0 | 0 | 2 | 0 | 0 | 0 | 1 | 0 | 1 | 1 | 0 | 0 | 0 | 0 | 0 | 1 | 0 | ? | 1 | 1 | 2 | 2 | 2 | 1 | 0 | 2 | 0 | 0 | 1 |
| *Africanomys major* | 0 | 0 | 1 | 0 | 0 | 0 | 0 | 2 | 0 | 0 | 0 | 1 | 0 | 1 | 1 | 0 | 0 | 0 | 0 | 0 | 1 | 0 | ? | 1 | 1 | 2 | 2 | 2 | 1 | 0 | 2 | 0 | 0 | 1 |
| *Africanomys minor* | 0 | 0 | 1 | ? | 0 | 0 | 0 | 2 | 0 | 0 | 0 | - | 0 | 1 | 1 | 0 | 0 | 0 | 0 | 0 | 1 | 0 | 0 | 1 | 1 | 2 | 2 | 2 | 1 | 0 | 2 | 0 | 0 | 1 |
| *Africanomys cf. solignaci* | 0 | 0 | 1 | ? | 0 | 0 | 1 | 1 | 0 | 0 | ? | ? | ? | 1 | 1 | 0 | 0 | 0 | 0 | 0 | 1 | 0 | ? | 0 | 0 | 2 | 2 | 2 | 1 | 0 | 2 | 0 | 0 | ? |
| *Proafricanomys libanensis* | 0 | 0 | 1 | ? | 0 | 0 | 1 | 1 | 0 | 0 | ? | ? | ? | 1 | 1 | 0 | 0 | 1 | 0 | 0 | ? | 0 | ? | 0 | ? | 2 | 2 | 2 | 1 | 0 | 2 | 0 | 0 | 1 |
| *Irhoudia robinsoni* | 1 | 0 | 2 | ? | ? | ? | ? | ? | ? | ? | ? | ? | ? | 1 | 2 | 1 | 0 | 0 | 1 | 0 | 2 | 0 | 0 | 1 | 2 | ? | ? | ? | 2 | 1 | 2 | 0 | 0&1 | ? |
| *Irhoudia bohlini* | 1 | 0 | 2 | ? | 0 | 0 | 0 | 2 | 0 | 0 | ? | ? | ? | 1 | 2 | 1 | 0 | 0 | 1 | 0 | 2 | 0 | 0 | 1 | 2 | ? | ? | ? | 2 | 1 | 2 | 0 | 0&1 | 0 |
| *Pellegrinia panormensis* | 2 | 1 | 2 | ? | - | - | - | - | - | - | - | - | - | ? | 2 | - | 1 | 0 | 1 | 1 | 2 | 1 | 0 | - | - | - | - | - | 2 | 0 | 3 | 0 | 1 | 0 |
| *Pectinator spekei* | 1 | 0 | 2 | 0 | 0 | 0 | 0 | 2 | 0 | 0 | 1 | 1 | 0 | 1 | 2 | 1 | 0 | 0 | 0 | 1 | 2 | 0 | 1 | 2 | 2 | 3 | ? | ? | 2 | 0 | 3 | 1 | 0 | 0 |
| *Ctenodactylus gundi* | 1 | 1 | 2 | 1 | 1 | 0 | 0 | 2 | 0 | 1 | ? | ? | ? | 1 | 2 | 1 | 1 | 0 | 1 | 1 | 2 | 1 | 0 | 2 | 2 | ? | ? | ? | 2 | 0 | 3 | 2 | 1 | 0 |
| *Ctenodactylus vali* | 1 | 1 | 2 | 1 | 1 | 0 | 0 | 2 | 0 | 1 | ? | ? | ? | 1 | 2 | 1 | 1 | 0 | 1 | 1 | 2 | 1 | 0 | 2 | 2 | ? | ? | ? | 2 | 0 | 3 | 2 | 1 | 0 |
| *Massoutiera mzabi* | 1 | 1 | 2 | 1 | 1 | 0 | 0 | 2 | 0 | 2 | - | - | - | 2 | 2 | - | 1 | 0 | 1 | 1 | 2 | 1 | 0 | 2 | 2 | - | - | - | 2 | 1 | 3 | 0 | 1 | 0 |
| *Felovia vae* | 1 | 1 | 2 | 1 | ? | 0 | 0 | 2 | 0 | ? | ? | ? | ? | 1 | 2 | 1 | 1 | 0 | 1 | 1 | 2 | 1 | 0 | 2 | 2 | ? | ? | ? | 2 | 1 | 3 | 0 | 1 | 0 |
